# Editorial: Phytochemicals in cancer prevention and therapeutics: Current advancement and future perspective

**DOI:** 10.3389/fphar.2023.1156197

**Published:** 2023-02-24

**Authors:** Subrata Kumar Pore, Suman Kumar Samanta, Bhaskarjyoti Gogoi, Eun-Ryeong Hahm

**Affiliations:** ^1^ Amity Institute of Molecular Medicine and Stem Cell Research (AIMMSCR), Amity University, Noida, Uttar Pradesh, India; ^2^ Life Sciences Division, Institute of Advanced Study in Science and Technology (IASST), Guwahati, Assam, India; ^3^ Department of Biotechnology, Royal Global University, Guwahati, Assam, India; ^4^ Department of Pharmacology and Chemical Biology, University of Pittsburgh, Pittsburgh, PA, United States

**Keywords:** phytochemicals, *Daemonorops draco* blume, aloe-emodin, hesperidin, flavokawain A, capsaicin, cancer therapy, chemoprevension

Many phytochemicals, naturally occurring small molecules isolated from living organisms, and their derivatives have been shown to act as potent candidates for cancer therapy and prevention due to their safety sketch. Accordingly they are of interest, as they could provide alternatives to the existing chemotherapeutic agents. Indian and Chinese traditional medicines have been using extracts from medicinal plants and herbs for thousands of years for the treatment of different diseases. Unfortunately, they are not considered as drugs according to the modern scientific definition. However, with the current advancements in organic and analytical chemistry, it is now possible to identify, isolate, and purify the active components of the extracts. Molecular pathway analysis implicated the roles of the active phytochemical ingredients in curing and inhibiting various diseases including cancer initiation, growth, and development. The anticancer effects of active phytochemicals involve pathways including the inactivation of carcinogens, the induction of apoptosis and cell cycle arrest, and the regulation of reactive oxygen species and immune system. A handful of different classes of phytochemicals like vinca alkaloids, camptothecin, podophyllotoxin, and taxanes are currently being used for different types of cancer, but with some adverse side effects. A few compounds from different classes like sulforaphane, curcumin, resveratrol, epigallocatechin gallate, berberine, etc. are in clinical trials. The aim of this Research Topic was to publish original research and review articles in this area to gain the current knowledge and future perspective of using phytochemicals in cancer therapy and prevention with reduced side effect. This Research Topic contains four original research articles and one review article.

Park et al. reported a traditionally used pain reliever in Korea, *Daemonorops draco* Blume (DD), also known as dragon’s blood, to have anticancer effects in acute myeloid leukemia (AML). DD extract depletes mitochondrial membrane potential through miR-216b by inhibiting c-Jun upon ER-stress response and leads to CHOP-dependent apoptosis in AML cells (Xu et al., 2016). Overall, the study showed the anticancer efficacy of the extract of DD, containing (2S)-7-hydroxy-5-methoxyflavan as major a metabolite in AML cells in an *in vitro* study. Pyroptosis, caspase-1-mediated programmed cell deaths, is an inflammatory pathway triggered by various pathological stimuli including cancer. Aloe-emodin (1,8-dihydroxy-3-hydroxymethyl-anthraquinone; AE), is an active component of various Chinese herbal medicinal plants. AE has been shown to exhibit anticancer effects *via* apoptosis, cell cycle arrest, and chemo-sensitization (Sanders et al., 2018). Li et al. showed that AE could induce mitochondrial dysfunction and pyroptosis through caspase 9/3/gasdermin E axis in a cervical cancer cell line. Authors emphasized that though both gasdermin D and E are involved in the activation of pyroptosis, AE acts through gasdermin E. However, gasdermin E is also expressed in normal tissues and immune cells. Though AE and its derivative emodin exhibit anticancer effects; drawbacks of these molecules are poor oral bioavailability, short half-life, and toxicities towards normal and immune cells.

Studies have shown the roles of hesperidin, a flavonoid phytochemical available in the skins of citrus species, to maintain blood pressure, cholesterol levels, to have anti-allergic, antiviral and anticancer effects (Muhammad et al., 2019). The anti-proliferative, and cancer prevention effects of this molecules have been reported in many cancers including liver, breast, prostate, and non-small cell lung cancer either as a single agent or in combination. Yao et al. showed that in Lewis lung carcinoma (LLC), hesperidin induces cellular senescence and inhibits invasion and migration by upregulating pinX1 protein, a telomerase inhibitor. Though the *in vitro* results were satisfactory, the antitumor effect of hesperidin *in vivo* animal model of LLC is limited even at higher dose mainly due to poor water solubility and bioavailability. However, at higher dose the compound was shown to be non-toxic for liver and kidneys. Suitable formulation and combination therapy may enhance the antitumor effects of this potent phytochemical.

Another manuscript in this Research Topic by Song et al. showed the efficacy of flavokawain A (FKA), a phytochemical with chalcone moiety extracted from kava plant, to inhibit the tumor initiation and stemness in prostate cancer. Dietary chemoprevention approach for prostate cancer after surgery or radiotherapy could be highly effective as the overall/disease free survival is considerably high with slow progression and long latency of the disease. In their previous manuscript, authors showed the chemo-preventive effect of dietary FKA in atransgenic adenocarcinoma of the mouse prostate model. FKA inhibits the high grade prostatic intraepithelial neoplasia lesions and distant organ metastasis (Li et al., 2015). Here, they reported that FKA could inhibit cancer stem cell (CSC)-initiated prostate cancer growth both *in vitro* and *in vivo* animal model as evidenced by the downregulation of several CSC-markers. Interestingly, enhanced expression of c-Mycis downregulated by FKA in prostate cancer bulk cells and CSC, isolated from xenograft tumors.

The review article in this Research Topic by Adetunji et al. discussed the roles of capsaicin, available in genus capsicum, in human cancer. The highly bioavailable capsaicin has numerous pharmacological effects on neuronal, cardiovascular, respiratory, urinary systems. Authors summarized the roles of capsaicin and its synthetic derivatives in many cancers such as breast, prostate, lung, etc. and showed that capsaicin is highly effective as a chemo-preventive agent due to its anti-mutagenic, antioxidant, and anti-inflammatory properties.
